# A New Medical Term

**Published:** 1890-07-26

**Authors:** 


### A New Medical Term.

It was said a long, long time ago that of making books there is no end. And certainly the same may be said of making medical terms. A good many books are of no use when they are made, and many new-fangled medical terms are positively mischievous. Dr. W. A. Hollis, physician to the Sussex County Hospital, is the last manufacturer of a new medical term—"chloremia." Dr. Hollis, while deprecating the unnecessary introduction of new words into scientific language, nevertheless thinks he is justified in offering the word chloremia as a substitute for the clumsy and inaccurate terms and paraphases which have hitherto served the purposes. Dr. Hollis refers to Hesiod, to Pindar, to Homer, to Sophocles, and to Thucydides, in order to show that *chloros* did not signify green, as we might suppose from the term "chlorosis" being applied to the malady popularly known as "green sickness." Chloros means rather a sallow or unhealthy skin, and was applied by the ancient writers named to signify various conditions, not only in a medical but in several other senses. Dr. Hollis defines *chloremia* to be a blood disorder, either idiopathic or associated with other ailments, consisting essentially of a greatly-diminished percentage of hæmoglobin, and of a variable decrease in that of the red particles. The malady principally attacks females, and nearly all his patients were between the ages of 18 and 25, the youngest being 16, and two only under 30. Most were domestic servants, not living in good sanitary conditions. A rheumatic family history was common to most patients. The prominent symptoms were breathlessness on exertion, often dry cough, palpitation, and frequently a *bruit du diable.* Œdema of the ankles was an occasional symptom. In the majority of cases there was some form of irregularity of menstruation. In the treatment no drug proved more serviceable than carbonate of iron, given after meals in drachm doses. In order to ascertain how far exposure to sunlight affected patients suffering from chloremia, several were placed in a bright and airy ward, without being given any medicine. The greatest increase of hæmoglobin under these circumstances was 2 per cent. in a week, while under the treatment with ferrous salts a similar improvement was noted in 24 hours. With reference to constipation being a cause of chloremia, Dr. Hollis remarks: "Moderate constipation—that is to say, when the bowels are naturally relieved every third or fourth day—was the rule among these patients . . . . . Some few cases of extreme irregularity of the bowels with obstinate constipation were met with, but these were by no means the most severe cases of chloremia." Contrary to the opinion expressed by Sir Andrew Clarke, Dr. Hollis does not think that autoinfection from retained fæcal matter is commonly productive of this blood disorder. On this we venture to observe that we should scarcely apply the term "moderate constipation" to the bowels being opened every third or fourth day; and that in our experience the condition which Dr. Hollis designates chloremia is very often associated with constipation, which, if relieved, will generally cure the chloremia. Now, there is no manner of doubt that the causes given by Dr. Hollis of chloremia are the causes of chlorosis; also that the symptoms given are those of chlorosis; thirdly, that the treatment given is that generally followed for chlorosis. Dr. Hollis, however, speaks of chlorosis as a form of chloremia. The only distinction, however, which we perceive is that the skin presents a tinge of green in the former, which it does not in the latter. It scarcely seems that this is sufficient to warrant our regarding the conditions as different diseases; any more than a variable tinge of a jaundiced skin, warrants our adopting the common expressions of "green" and "yellow" jaundice, as distinctive of different diseases. Perhaps there may be more said in favour of Dr. Hollis' new term chloremia than of many other new terms which have been invented, but we have already a generic term for blood disorders in *anæmia*, which, although not perhaps free from objection, is established, and suits tolerably well.

Colonel Ingersoll has taken a new departure, and has been attacking vivisectionists as "scientific assassins." He says he knows that all the torture has been useless, and that all the knowledge could have been attained by the dissection of the dead, or at least of animals perfectly under the influence of ether. Colonel Ingersoll then knows a good deal. Somehow one scarcely expected this depth of feeling from the exquisitely-refined author of "Some Mistakes of Moses."

